# Calpain Dysregulation in Alzheimer's Disease

**DOI:** 10.5402/2012/728571

**Published:** 2012-10-16

**Authors:** Adriana Ferreira

**Affiliations:** Department of Cell and Molecular Biology, Feinberg School of Medicine, Northwestern University, 303 E. Chicago Avenue, Ward 8-140, Chicago, IL 60611, USA

## Abstract

Alzheimer's disease (AD) is characterized by the presence of senile plaques and neurofibrillary tangles in the neocortex and hippocampus of AD patients. In addition, a marked decrease in synaptic contacts has been detected in these affected brain areas. Due to its prevalence in the aging population, this disease has been the focus of numerous studies. The data obtained from those studies suggest that the mechanisms leading to the formation of the hallmark lesions of AD might be linked. One of such mechanisms seems to be the dysregulation of calcium homeostasis that results in the abnormal activation of calpains. Calpains are a family of Ca^2+^-dependent cysteine proteases that play a key role in multiple cell functions including cell development, differentiation and proliferation, axonal guidance, growth cone motility, and cell death, among others. In this paper, we briefly reviewed data on the structure of these proteases and their regulation under normal conditions. We also summarized data underscoring the participation of calpains in the neurodegenerative mechanisms associated with AD.

## 1. Introduction

Alzheimer's disease (AD) is the most common cause of dementia in the aging population. This disease develops over time and leads to significant cognitive deficits affecting memory, insight, judgment, abstraction, and language functions [1]. AD affects more than 5 million people in the United States and this number is projected to rise to 35 million by 2050 [2, 3]. This estimate underscores both the scope of this health care issue for the society as a whole and the need for the development of therapeutic options for these patients. 

The diagnosis of this neurodegenerative disease relies on the presence of senile plaques and neurofibrillary tangles in affected brain areas at autopsy. These AD hallmark lesions are the results of the pathological deposition of proteins normally present throughout the brain. Senile plaques are composed of extracellular deposits of beta-amyloid (A*β*) derived by proteolytic cleavage from the amyloid precursor protein (APP) [4–10]. Neurofibrillary tangles, on the other hand, are intracellular bundles of self-assembled tau proteins [11–38]. The formation of both senile plaques and neurofibrillary tangles is associated with progressive and irreversible degeneration of neuronal processes and the loss of synaptic connections [39–50]. 

 Initially, multiple studies focused on defining the characteristics of AD and on the analysis of the composition of senile plaques and neurofibrillary tangles. More recently, data have been obtained on the molecular mechanisms that link the formation of these lesions and underlie neurodegeneration and cell death in AD and related disorders. Calpains seem to play a key role in such mechanisms. Calpains are Ca^2+^-dependent proteases in which activity is dysregulated in AD and other neurodegenerative diseases [51–54]. A growing body of evidence suggests that the abnormal activation of calpains might modulate not only the formation of senile plaques and neurofibrillary tangles but also the development of synaptic pathology in AD. These data reviewed below position calpains at the crossroads of the mechanisms involved in the formation of the main pathological alterations associated with AD. Furthermore, these findings underscore the importance of calpains as potential targets to block the activation of the signaling cascades leading to degeneration in AD. In turn, this information could be applied to the design of therapeutic options and prevention strategies for this devastating disease. 

 In this review, we first summarized data on the properties of calpains and the mechanisms underlying their activation. Then, we reviewed findings on the dysregulation of calpain in the context of AD and its deleterious consequences for the morphology and function of affected brain areas. Finally, we examined the effects of experimental manipulations that could prevent such effects. 

## 2. The Calpain Family

 Calpains constitute a family of Ca^2+^-dependent cysteine proteases involved in multiple and very diverse cell functions including cell development, proliferation, and differentiation, cell motility, growth cone motility and guidance, apoptosis, learning, and memory, among others [51, 52]. Originally, two members of this family were identified: calpain 1 (*μ*-calpain) and calpain 2 (m-calpain), also known as conventional or classical calpains [51]. More recently, the other 14 members of the calpain family have been identified in mammals [51, 55]. In contrast to classical calpains that are ubiquitously distributed, the expression of some of these unconventional or nonclassical calpains is tissue specific. For example, calpain 3a and calpain 8 are mainly present in skeletal and smooth muscle cells, respectively. Calpain 11, on the other hand, is highly expressed in testes, while calpain 13 is concentrated in the lung and skin. Others, like calpains 5, 7, 9, and 10 have more widespread distributions [51]. None of these new members of the calpain family are highly expressed in the central nervous system (CNS) [51]. Thus, we will focus this review on the conventional calpains. 

 Both calpain 1 and calpain 2 are heterodimers composed of a large catalytic subunit (~80 kDa) and a small regulatory subunit (~30 kDa). The amino acid sequence of these subunits is highly conserved in mammals [51]. Based on this sequence, the catalytic subunit has been divided into four distinct domains (reviewed in [51]). Domain 1 corresponds to the N-terminal region of this subunit and undergoes autolysis upon Ca^2+^ binding. Domain 2 contains a catalytic unit formed by Cys, His, and Asn residues. This unit is characteristic of cysteine proteases like papain or cathepsin [56]. Domain 3 links the Ca^2+^ binding and the catalytic domains and regulates calpain activity. It might also bind phospholipids [57–59]. Domain 4 has partial homology with calmodulin and contains several EF-hand calcium-binding motifs [60]. The regulatory subunit can be divided into two domains, a calmodulin-like domain and a glycine-rich domain [61]. 

 Calpain 1 and calpain 2 are highly expressed throughout the CNS. While both proteases are present in neurons and glial cells, their relative abundance differs. Calpain 1 is more abundant in neurons, and calpain 2 is prominent in glial cells [62]. Analysis of the subcellular localization of these enzymes demonstrated that calpain 1 is concentrated in the cell bodies and is also detected in the processes extended by central neurons, although at lower levels. In addition, calpain 1 is present at synaptic sites. Within the presynaptic element, calpain 1 is enriched between synaptic vesicles. Calpain 1 immunoreactivity has been also detected in postsynaptic densities as well as in dendritic spines [63]. Calpain 2 immunoreactivity is mainly present in white matter and in myelinated axons. Astrocytic processes are also enriched in calpain 2 [62, 63].

Calpains cleave proteins generating large fragments. Initially, it was thought that these proteases' cleavage sites have a Leu or a Val residue in the P2 position [64]. More recently, it has been shown that the cleavage site specificity is determined by conformation rather than amino acid sequence [65–68]. The specificities of the substrates for both calpains are very similar but not identical. *In vitro* studies have shown that more than 100 proteins could be cleaved by calpains. Calpain substrates include cytoskeletal proteins (cadherin, catenin, desmin, dystrophin, gelsolin, filamin, fodrin, microtubule-associated proteins MAP 1 and MAP 2, neurofilament proteins, spectrin, tau, talin, troponin, tubulin, vimentin, and vinculin), signal transduction proteins (calcium/calmodulin-dependent protein kinase, epidermal growth factor (EGF) kinase, pp60, protein kinase C, calcineurin, and caspases 3, 7, 8, 9, 12, and 14), synaptic proteins (dynamin 1, postsynaptic density (PSD) 95, N-methyl-D-aspartic acid (NMDA) glutamate receptors, and metabotropic glutamate receptor GluR1), and transcription factors (p53), among others [51, 53].

 Calpain activation is tightly regulated to prevent massive proteolytic activity in the cell. The main regulator of this activity is Ca^2+^ [51]. The binding of Ca^2+^ to either calpain results in conformational changes that initiate their proteolytic activity. While calpain 1 is activated in the presence of micromolar concentrations of Ca^2+^, calpain 2 requires millimolar concentrations [51]. The Ca^2+^ requirement for either calpain is significantly higher than the concentration of this ion in living cells. This fact has prompted a series of studies on potential mechanisms that could lead to a decrease in the Ca^2+^ requirement for the activation of these proteases in living cells. *In vitro* studies have shown that the interaction of calpains with a series of activator molecules lowers the concentration of Ca^2+^ required to initiate the activation of these proteases. Among them, phospholipids (i.e., phosphatidylinositol) are the most studied. However, the phospholipid to calpain molar ratio required for lowering the Ca^2+^ requirement for either calpain activation is not likely to be achieved in the cellular context [69–71]. Nevertheless, it cannot be ruled out that higher concentrations of Ca^2+^ are achieved in limited cellular areas where Ca^2+^ is not easily detectable by available methods. In turn, these local micromolar or millimolar Ca^2+^ concentrations could trigger calpain activation in distinct subcellular domains.

 In addition to Ca^2+^, calpastatin has a key role in the regulation of calpain. Calpastatin, a heat-stable protein ranging from ~70 to ~140 kDa of apparent molecular weight depending on the cell type, is considered a specific endogenous inhibitor of calpains [72]. It is ubiquitously expressed and has a widespread cytosolic distribution [73]. Immunocytochemical analysis showed calpastatin immunoreactivity throughout the dendritic tree in pyramidal neurons and Purkinje cells. On the other hand, weak calpastatin labeling was detected in glial cells [74]. The calpastatin molecule contains four inhibitory units [75–77]. Each of these units binds to one calpain molecule [75–77]. Therefore, the ratio calpain/calpastatin plays a key role in the regulation of calpain activity [78–80]. The inhibitory effect of calpastatin requires Ca^2+^-dependent high-affinity binding to three sites of calpain [51].

 Phosphorylation might be also involved in the regulation of calpain activity. Both calpains can be phosphorylated in multiple sites of the catalytic subunit [51]. The kinases responsible for the phosphorylation of some of these sites have already been identified. Thus, two of those sites seem to be phosphorylated by protein kinase C (PKC), two by protein kinase A (PKA), two by calmodulin kinase II, one site by casein kinase I, and another by protein kinase G [51]. Although the role of the phosphorylation of each site in calpain activation has not been completely elucidated, data suggest that phosphorylation mediated by mitogen-activated kinases (ERK/MAP kinases) activates these proteases [81]. On the other hand, phosphorylation by PKA may decrease their activity [82, 83].

## 3. Dysregulation of Calpain Activity in AD and Related Disorders

As briefly reviewed above, calpain activation is a tightly regulated process to prevent deleterious consequences of massive proteolytic activity. These regulatory mechanisms seem to decline with aging resulting in an increased calpain activity [84–87]. In addition, these proteases seem to be abnormally activated under pathological conditions. Thus, several studies have reported significantly increased activation of calpains in AD brains [88–94]. The hyperactivation of calpain in the context of AD is the result of several factors including enhanced intracellular Ca^2+^ concentration and decreased calpastatin levels. Experiments performed using an AD culture model system showed that oligomeric A*β* induced a significant (5-fold) and instantaneous rise in Ca^2+^ in hippocampal neurons [95–97]. These studies also addressed the source of the Ca^2+^ influx leading to calpain activation in the context of AD taking advantage of specific blockers of Ca^2+^ release from the endoplasmic reticulum, the major source of intracellular Ca^2+^, and BAPTA, a chelator of extracellular Ca^2+^ [98]. The data obtained showed that A*β* induces calpain activation by enhancing extracellular Ca^2+^ influx [95–97]. The mechanisms underlying the regulation of the enhanced Ca^2+^ influx and calpain activation in AD have also been studied. Both NMDA receptors and voltage-gated calcium channels (VGCC) have been implicated in such regulation in central neurons [99–101]. Specific NMDA receptor inhibitors, MK801 [102], and the FDA approved NMDA receptor antagonist memantine [103] significantly attenuated the initial A*β*-induced increase of Ca^2+^ and blocked A*β*-induced calpain activation in cultured hippocampal neurons [95–97]. In contrast, nimodipine, an L-type VSCCs blocker [104], did not decrease the A*β*-induced activation of calpain [95–97]. These data suggest that NMDA receptors play an important role in mediating the sustained Ca^2+^ influx and enhanced calpain activity induced by aggregated A*β*. Furthermore, these studies suggested that factors that affect NMDA receptor-mediated Ca^2+^ influx could modulate calpain activation and its deleterious effects in central neurons. Cholesterol seems to play such a role. Recently, it has been shown that cholesterol regulates calpain activity in the context of AD [105]. Cholesterol is a risk factor for AD. A population-based study demonstrated that not only high cholesterol levels but also moderately elevated cholesterol levels in midlife represent a significant risk factor for AD [106, 107]. Interestingly, cholesterol has been implicated in the susceptibility of cells to Ca^2+^ influx [108–111]. Thus, elevated membrane cholesterol actually increased the susceptibility of cells to A*β*-induced elevation in Ca^2+^ influx leading to cell death via a calpain-dependent mechanism [111, 112]. By regulating the NMDA receptor content and changing their localization in membrane microdomains at the synaptic sites, cholesterol modulates the ability of A*β* to induce Ca^2+^ influx, leading to calpain activation in hippocampal neurons [111, 112].

Calpastatin also seems to play an important role in the regulation of calpain activation in AD. Thus, it has been shown that calpastatin is markedly depleted in the cortex of AD brains at late stages of the disease as compared to age-matched controls. Focal areas of calpastatin depletion have been also detected along dystrophic neurites at early stages of AD [74]. On the other hand, no changes in calpastatin levels were detected in neurons less susceptible to neurodegeneration in AD like Purkinje cells [113]. This decrease in calpastatin levels is the result of the proteolytic activity of caspases and calpain [74]. In turn, the decrease in the ratio calpastatin to calpain causes calpain hyperactivation perpetuating this cellular deleterious effect [79, 113–115]. 

## 4. The Role of Calpain in the Generation of Pathological Manifestations of AD

**Figure 1:**
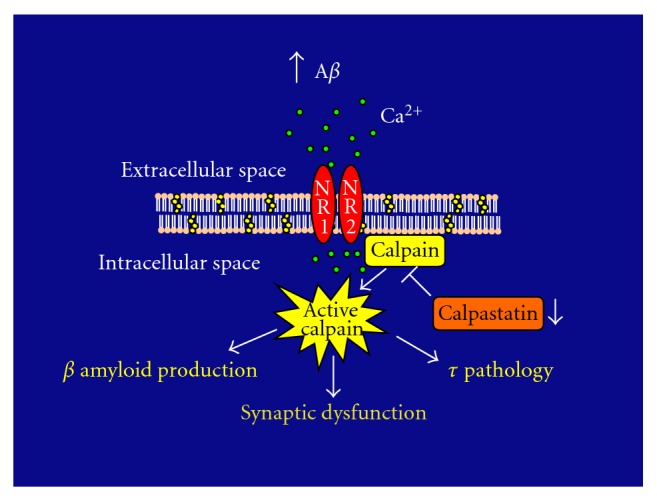
In the context of Alzheimer's disease, increased levels of beta-amyloid (A*β*) induce calcium (Ca^2+^) influx through NMDA receptors (NR1 and NR2) in hippocampal neurons. This Ca^2+^ influx and a decrease in calpastatin levels result in the dysregulation of calpain activity leading to the cleavage of a series of proteins involved in the formation of senile plaques and neurofibrillary tangles as well as in synaptic dysfunction.

The data reviewed above provide insights into the abnormal calpain activation in AD brains. These findings have been complemented with immunocytochemical studies on the distribution of calpain in AD. These studies have shown intense calpain immunoreactivity in dystrophic neurites associated with neurofibrillary tangles, neuritic plaques, and neuropil threads in the hippocampal region and entorhinal cortex of subjects suffering from AD and other tauopathies [116–118]. Based on this information, it was hypothesized that the enhanced calpain activity observed in AD brains plays an important role in the formation of senile plaques, neurofibrillary tangles, and synaptic dysfunction in the context of AD (Figure 1). Senile plaques are mainly composed of A*β*_1–40_ and A*β*_1–42_ peptides [4]. These peptides are the result of the sequential proteolytic cleavage of APP by two proteases. First, *β*-secretase (*β*-site APP-cleaving enzyme (BACE 1)) cuts APP at the N-terminus of the A*β* domain leaving a 99-amino-acid-long C-terminal fragment. Then, the *γ*-secretase complex (presenilin 1, presenilin 2, nicastrin, APH1, and PEN2) cuts the C-terminus to generate A*β* peptides of 38 to 43 amino acids in length [5, 119–121]. Of these peptides, A*β*_1–42_ is regarded as the main pathogenic species [122–130]. Therefore, increased levels of BACE 1 could lead to enhanced production of A*β*, its aggregation, and consequently the activation of signaling pathways associated with the neurodegenerative process. This seems to be the case in AD patients. Thus, a 2-fold increase in BACE1 levels has been detected in AD brains [127–129]. Recently, it has been shown that the calpain/calpastatin system can modulate the pathological deposition of A*β*. Calpain activation increases the levels of BACE1 in a transgenic mouse model of AD [130]. In addition, the bidirectional regulation of the activation of the calpain/calpastatin system and A*β* metabolism has been established [131]. Using transgenic mice overexpressing APP, these authors showed that calpastatin deficiency enhanced calpain activation, A*β* production, and increased mortality. In turn, the increased levels of A*β* lead to enhanced Ca^2+^ influx and increased calpain activation [131].

Senile plaques are often surrounded by dystrophic neurons that contain tau aggregates known as neurofibrillary tangles. Many studies have focused on the composition and the mechanisms underlying the formation of tau aggregates. Those studies have identified two types of tau filaments in the neurofibrillary tangles: straight and paired helical filaments. Hyperphosphorylated forms of tau form both types of filaments [132–142]. Tau hyperphosphorylation has been attributed to the increased activity of several kinases, including cyclin-dependent kinase 5 (CDK5), glycogen synthase kinase *β* (GSK*β*), and the mitogen-activated protein kinase (MAPK) in hippocampal neurons [138, 143–145]. Tau phosphorylation can be modulated by calpain. Thus, it has been shown that calpain cleaves the inhibitory domain of GSK3 generating two fragments of 40 and 30 kDa. This cleavage enhanced activity of the kinase [146]. Calpains also modulate the activity of CDK5. Physiologically, CDK 5 is activated by p35 and its cleaved product p25. The latter has a longer half life than p35 and therefore it is a more potent activator of CDK5. The cleavage of p35 to p25 is mediated by calpain [147–149]. In addition, calpains activate ERK/MAP kinases [150]. Experiments using calpeptin, a cell permeable calpain inhibitor, blocked both ERK 1 and ERK 2 activation. Conditions that blocked the activation of these kinases induced a decreased in tau phosphorylation and resulted in enhanced neuronal survival in the presence of A*β* [136].

Besides tau phosphorylation, calpain activation might play a role in tau-mediated neurodegeneration by inducing tau cleavage. *In vitro* studies have shown that both fetal and adult tau isoforms are rapidly proteolyzed by calpains [151–153]. On the other hand, tau present in paired-helical filaments is considerably more resistant to proteolysis by these proteases [153–156]. These findings suggest that phosphorylation might regulate the susceptibility of tau to calpain-mediated cleavage [153]. Support for this hypothesis was obtained using cultured hippocampal neurons treated with okadaic acid. Tau obtained from cultured neurons incubated in the presence of this phosphatase inhibitor was more resistant to calpain cleavage than tau extracted from nontreated control neurons [90]. Nevertheless, the effect of phosphorylation on calpain-mediated tau cleavage seems to be complex. This effect might depend on the site phosphorylated and/or the extent of phosphorylation under pathological conditions since highly phosphorylated fetal isoforms are readily cleaved by calpain [153]. 

Calpain-mediated tau cleavage seems to play an important role under neurodegenerative conditions [157–164]. It has been shown that calpain activation results in the generation of several N-terminal tau fragments. One of such tau fragments, of ~20 kDa of apparent molecular weight, has been detected in mitochondria present in synaptosomal fractions obtained from AD brains [158]. The levels of this tau fragment are partially reduced when cultured neurons are treated with a calpain inhibitor [158]. The presence of this fragment could impair mitochondria function. It has been shown that tau fragments containing the 26–44 tau amino acids affect mitochondria oxidative phosphorylation acting at the level of the adenine nucleotides translocator contributing to synapse dysfunction [159]. 

A smaller tau fragment generated by calpain cleavage has been detected in mature hippocampal neurons treated with aggregated A*β* oligomers [160, 161]. A*β*-induced tau cleavage mediated by calpain 1 leads to the generation of a neurotoxic 17 kDa tau fragment (tau 45–230) in cultured hippocampal neurons [160, 161]. This fragment has also been detected in the neocortex of AD brains as well as in brain areas affected by other tauopathies [90]. Furthermore, when expressed in neuronal and nonneuronal cell types or in an *in vivo Drosophila* model system, the 17 kDa tau fragment produced cell death in the absence of A*β* oligomers [160, 161, 163]. These data provided strong evidence for an important role of calpain 1 and the generation of the 17 kDa tau fragment in the progression of A*β*-mediated neurodegeneration. It is worth noting that calpain 2 activation cleaves tau generating a smaller tau fragment that lacks neurotoxic effects in central neurons [164]. 

In addition to the presence of senile plaques and neurofibrillary tangles, AD brains are characterized by a decrease in synaptic contacts. This synaptic loss seems to be the best morphological correlate of the functional deficits observed in the mid to late stages of AD [39, 40]. Although no significant decline in synapse number has been detected in the earliest stages of the disease, a stage of synaptic dysfunction seems to precede frank synapse loss [41, 46, 165]. By cleaving proteins in the presynaptic terminals and/or the postsynaptic elements, calpain could induce profound functional changes in affected hippocampal neurons [166].

Changes in proteins involved in synaptic vesicle biogenesis and/or recycling at synaptic terminals are responsible, at least in part, for the synaptic dysfunction detected in AD [43]. Recently, data obtained using culture and animal models of AD showed that A*β* induced a significant reduction in dynamin 1 levels that preceded synapse loss. Dynamin 1 is a neuron-specific mechanochemical GTPase highly enriched in presynaptic terminals. Dynamin 1 pinches off synaptic vesicles, freeing them from the membrane and allowing them to reenter the synaptic vesicle pool to be refilled for future release [167, 168]. Decreased levels of this protein lead to the depletion of synaptic vesicles and the accumulations of invaginated pits at presynaptic membranes adjacent to the synaptic clefts [95–97, 169, 170]. The A*β*-induced decrease in dynamin 1 is a result, at least partially, of calpain-mediated proteolysis [95–97, 171]. 

Calpain dysregulation could also affect proteins in the postsynaptic element. Thus, it has been shown that calpain activation results in the breakdown of several proteins leading to changes in the ultrastructure of the postsynaptic density (PSD). These changes in the PSD are accompanied by the rapid cleavage of PSD-95 and the NMDA receptor subunits NR1 and NR2A and 2B [172–175]. This cleavage is regulated by the tyrosine kinase Fyn phosphorylation of the NR2B subunit [176]. Calpain has been shown to truncate also mGluR1 exacerbating NMDA-mediated neurotoxicity [176–178]. 

An additional mechanism by which calpain activation can result in synaptic dysfunction involved the cleavage of PKA leading to a decrease in both the regulatory and the catalytic subunits of this kinase. The decrease in PKA activity attenuates CREB activation impairing memory [179, 180]. 

## 5. Future Directions and Concluding Remarks

Taken together, the data reviewed above strongly suggest that calpains play an important role in the neurodegeneration mechanisms underlying AD in the aging population. Based on these data, it has been tempting to speculate that calpain inhibition could be a useful tool to prevent neurodegeneration in this disease. To obtain further insights into the beneficial effects of blocking calpain activation, several studies have been conducted using experimental approaches to either suppress the expression of these proteases or prevent their abnormal activation. Early studies using gene deletion of the calpain regulatory subunit by homologous recombinant techniques showed that the depletion of these proteases is embryonic lethal [181]. Later, studies have been performed using specific antisense oligonucleotides. The data obtained showed that indeed the suppression of calpain 1 expression by means of these specific probes prevented oxidative stress-induced cell injury in human hepatic cancer cell lines [182]. Other studies have assessed the effects of calpain inhibitors on cell death in culture model systems of AD. Leupeptin and E64 calpain inhibitors had protective effects in those cultures [160, 183–186]. The idea that calpain could be a potential therapeutic target in neurodegenerative diseases has been reinforced by studies performed *in vitro* and in culture using the calpain inhibitor MDL 28170. Those studies suggested a protective effect of calpain inhibitors against excitotoxicity [178, 187, 188]. Unfortunately, the potential use of known calpain inhibitors as therapeutic tools in AD is limited due to their low cellular penetration, poor selectivity, and kinetics. Recently, A-705053, a novel calpain inhibitor with improved pharmacokinetics, has been characterized [189]. This benzoylalanine-derived ketoamide is capable of inhibiting calpain in nanomolar concentrations and has improved oral bioavailability, water solubility, and metabolic stability [189]. This calpain inhibitor is highly effective in preventing calpain-mediated cleavage of dynamin 1 and tau in cultured hippocampal neurons. This inhibitor is effective not only when added prior to the A*β* treatment but also when added simultaneously with A*β* and even when added after A*β* has triggered the neurodegeneration process [171]. The calpain inhibitor A-705253 also prevents A*β* oligomer-induced neurodegeneration of the nucleus basalis magnocellularis [190]. These experiments raised the possibility that more potent calpain inhibitors could have beneficial effects even at late stages of AD. Moreover, initial studies using calpain inhibitors in mouse and rat models of AD showed an encouraging recovery of cognitive function in these animals when they were treated with calpain inhibitors at an early age [186, 190]. Together, these data underscore the potential importance of calpain inhibitors as promising therapeutic tools in AD and related neurodegenerative diseases.

 In summary, the data briefly reviewed above provide strong support for the role of calpains in AD. They also highlight the tantalizing possibility that these proteases could serve as targets for the development of therapeutic interventions for this disease, one of the main challenges for decades to come in AD research.
